# Deep Multimodal Learning for the Diagnosis of Autism Spectrum Disorder

**DOI:** 10.3390/jimaging6060047

**Published:** 2020-06-10

**Authors:** Michelle Tang, Pulkit Kumar, Hao Chen, Abhinav Shrivastava

**Affiliations:** 1 Science, Math, and Computer Science Magnet Program, Montgomery Blair High School, Silver Spring, MD 20901, USA; mtang@umiacs.umd.edu; 2 Institute for Advanced Computer Studies, University of Maryland College Park, College Park, MD 20742, USA; pulkit@cs.umd.edu (P.K.); chenh@cs.umd.edu (H.C.)

**Keywords:** deep learning, multimodal learning, convolutional neural networks, autism, fMRI

## Abstract

Recent medical imaging technologies, specifically functional magnetic resonance imaging (fMRI), have advanced the diagnosis of neurological and neurodevelopmental disorders by allowing scientists and physicians to observe the activity within and between different regions of the brain. Deep learning methods have frequently been implemented to analyze images produced by such technologies and perform disease classification tasks; however, current state-of-the-art approaches do not take advantage of all the information offered by fMRI scans. In this paper, we propose a deep multimodal model that learns a joint representation from two types of connectomic data offered by fMRI scans. Incorporating two functional imaging modalities in an automated end-to-end autism diagnosis system will offer a more comprehensive picture of the neural activity, and thus allow for more accurate diagnoses. Our multimodal training strategy achieves a classification accuracy of 74% and a recall of 95%, as well as an F1 score of 0.805, and its overall performance is superior to using only one type of functional data.

## 1. Introduction

Autism spectrum disorder (ASD) is a lifelong neurological condition characterized by repetitive, restricted behavior as well as limitations in communication and social abilities, manifesting in a wide range of sensorimotor deficits [1]. Diagnostic processes for ASD are based on the assessment of social behaviors and language skills; however, there is limited understanding of the neural patterns behind the spectrum of autism behavior and the severity of the disease [2]. Recently, noninvasive brain imaging techniques have allowed for a better understanding of the neural circuitry underlying neurodeficiency disorders and their associated symptoms, such as ASD and its behavioral deficits. Specifically, functional magnetic resonance imaging (fMRI) allows physicians and medical experts to visually evaluate the functional characteristics or properties of a brain. This radioimaging procedure is able to assist neuroscientists in acquiring valuable and precise information on different neurological disorders [3]. For instance, in the diagnosis of ASD, instead of solely relying on observation and speaking with the patient, physicians analyze neuroimages for anomalies in brain activity, helping them more efficiently and precisely identify differences in their patients’ neural pathways. Identifying the neural patterns of activation for ASD and associating these patterns with physiological and psychological features will aid scientists and medical practitioners in gaining more insight into the etiology of mental disorders [4].

**Computational diagnosis:** However, even with high-quality images, manual analysis is inefficient and at risk of human error, due to the variation between both doctors and patients. In an effort to make this process more objective, machine learning and computer vision techniques have frequently been applied in medical imaging problems to construct computer-assisted diagnosis systems [5]. State-of-the-art neuroimaging analysis uses activation maps, alternatively known as correlation matrices, which are constructed from the radiology scans. Activation maps provide information regarding the interaction between different areas of the brain by describing the relationship between their corresponding neural activity [6]. In doing so, the activation maps allow scientists to localize abnormal neural activity to specific regions of the brain and focus on those regions when manually analyzing the scans for anomalies.

Traditional machine learning in computer vision relies heavily on features pre-selected by medical experts. Early machine learning algorithms, e.g., support vector machines (SVM), k-nearest neighbors (KNN), decision trees, etc., analyze raw image data without any learning of hidden representations. On the other hand, deep learning (DL) algorithms in computer vision has shown significant advantage in capturing hidden representations and automatically extracting features from the given image data. Based on artificial neural networks structures, DL algorithms have multiple stages of non-linear feature transformation, which leads to hierarchical representations from pixels to edge, motif, part, and the entire object. State-of-the-art deep learning algorithms include convolutional neural networks (CNNs), recurrent neural networks (RNNs), long short term memory (LSTM) networks, generative adversarial networks (GANs), etc., which do not require manual processing or feature selection on raw data.

### Related Work

The Autism Brain Imaginge Data Exchange (ABIDE) is a public, anonymized neuroimaging dataset that has been analyzed extensively in the past using various different techniques. Nielsen [7] analyzed ABIDE data to classify autism versus control subjects based on brain connectivity measurements. The study replicated earlier methods and implemented them on point-to-point connectivity data, and achieved roughly 60% accuracy with whole-brain classification.

**Connectivity maps.** The most common approach to fMRI analysis focuses on ROI mean time series data, since it is a representation of brain activity in distinct regions of the brain. Prior to analysis, pair-wise correlations are calculated between ROIs to form a functional connectivity matrix—each element of the matrix is a Pearson correlation coefficient representing the level of co-activation between the signals in the two ROI based on the time series data. The resulting matrix, also known as an activation map, is reshaped into a feature vector and then used as input into a classification algorithm [8,9].

**Full-brain connectivity fingerprints.** There are several arguments against exclusively using ROI time series data to analyze fMRI scans. The original form of each functional scan is a complete 3D scan of the brain across time (i.e., 4D matrix); thus, averaged ROI signal intensities only offers a partial representation of the fMRI scan, since by condensing the correlation matrix, one effectively loses the spatial structure of the connectome [10]. Khosla et. al. (2017) [9] proposed an alternative to relying exclusively on ROI data—instead of only computing the correlation between each region’s signal intensities, they construct a full-brain connectivity “fingerprint” consisting of correlation coefficients between voxels from the fMRI scan and signal intensities from ROIs, incorporating both the spatial information offered by the original functional image volume along with the averaged time series data. The connectivity fingerprint is then used as input into a simple CNN architecture [8].

**Multimodal learning.** Computer vision and deep learning have been used extensively to achieve automatic disease diagnosis with medical imaging analysis [11,12]. Specifically, many machine learning problems involve the analysis of different forms of data to arrive at a result; as part of the classification problem, it is important to understand the relationship between these different data types, or modalities of data. Zhang et. al. (2018) [13] use generative adversarial networks (GANs) to translate between computed tomography (CT) and magnetic resonance imaging (MRI) data. Fukui et. al. (2016) [14] propose a new method for multimodal compact bilinear (MCB) pooling by computing the outer product of the different feature vectors from multiple modalities. MCB is based on previous compact bilinear pooling methods and allows for a more expressive and also efficient way of constructing a joint representation of both inputs [15]. The model was used to solve visual question answering (VQA) and visual grounding problems and outperforms state-of-the-art methods.

**Our Approach.** Previous studies on functional neuroimaging analysis use only one type of activation map in their evaluation [8,9,16]. In this study, we propose to include both activation maps in our analysis, and that by using two modalities instead of one, we will be able to create a more accurate and holistic representation of the functional imaging data, and thus achieve more accurate diagnoses. To do so, we propose a model architecture capable of analyzing both types of activation maps by combining different deep learning networks. Specifically, one of the inputs is the ROI time series activation map as described above, constructed by computing the correlation matrix between all pairs of ROI, and the second is the fMRI × ROI activation map. The pooling technique used in our model is a concatenation of the two feature vectors, which is then used as input to four fully-connected layers before the network outputs its prediction. The resulting classification system is a multimodal model, an automated disease classification system that uses two types of activation maps to predict whether the given patient is healthy or has autism. With both input modalities, the resulting diagnosis system is both more accurate and offers a more complete understanding of the functional data, and provides a powerful aid to physicians during the diagnostic process.

The main contributions of our method are highlighted as follows:

1. We propose an end-to-end deep neural network-based multimodal architecture that incorporates features from two different types of modalities. Our proposed architecture incorporates all connectomic information from the functional imaging data, which allows for a more comprehensive and holistic functional image analysis.
2. By incorporating both types of activation maps from the functional data, the overall performance of the classifier is improved and thus creates a more powerful diagnostic tool to assist doctors. Moreover, we also provide ‘visual explanations’ of the regions in fMRI images that our model utilizes to make diagnosis predictions. We believe this visualization offers transparency on the model and is crucial in providing effective assistance to the diagnostic process.

The remainder of this paper is organized as follows: in Section 2, we present the dataset and imaging modalities that were analyzed in our study, and our methods for analysis, including the preprocessing pipeline and network architectures; in Section 3, we discuss the details behind the training and testing of our constructed models; in Section 4, we present the results and performance metrics of each model and discuss them with respect to other publications in the field; and we present our conclusions and future work in Section 5.

## 2. Methods and Data

### 2.1. Data

In this study, we focus on the analysis of functional magnetic resonance imaging, or fMRI scans. The purpose of these images is to track changes in blood oxygen level-dependent (BOLD) signal in the brain across a period of time. Functional images are thus four-dimensional, where the dimensions (H,W,D,T) represent the height, width, depth, and time dimensions of the image volume, respectively [3]. fMRI scans can also be represented as regions of interest (ROI) mean time series data [17,18], which is constructed from the fMRI scan after preprocessing, and can be considered of as an abridged or summarized version of the complete multidimensional fMRI image volume. The ROI time series data is constructed by first segmenting the original fMRI scan functionally homogeneous regions (ROI), and then taking the average BOLD signal from each area. These ROIs are typically segmented using a functional parcellation brain atlas [19]; different atlases “segment” the full brain scan into different ROIs. For the purposes of our research, we chose to use the Automated Anatomical Labeling (AAL) atlas. After the regions have been specified, the BOLD signal intensities of each voxel are averaged across all voxels enclosed in the region, to return one intensity value for each time frame. The result is a T-dimensional vector for every ROI, where T represents the number of seconds for which the signal was measured. Thus, for each subject, one ROI mean time series volume is a 2D matrix with dimensions (N,T), where *N* represents the number of ROIs [8].

However, the BOLD signal measured by fMRI images represents changes in the blood oxygenation of brain tissue, and are not equivalent to neural activity [20]. Thus, it is not appropriate to directly use the fMRI scan of BOLD signals nor the ROI time series data as input for analysis. Instead, correlations between the time series data must be computed, either between voxels and ROIs or only pairs of ROIs—these correlations serve as the two different features used in our model. The resulting output from this step is a correlation matrix or activation map, which indicates points in the scan where there is neural activity.

This study was conducted on the ABIDE dataset [21], a multi-site open-access MRI study containing anonymized patient neuroimaging data. Data from the first phase of the study (ABIDE-I) was used in our analysis, which consisted of fMRI images and regions of interest (ROI) time series signals from both control and disease groups. The original ABIDE-I dataset consists of 1112 subjects; in this study, 77 subjects were removed from consideration due to low quality or missing scans. This yielded a total of 1035 subjects, with 505 individuals diagnosed with ASD and 530 typical controls, from 17 sites.

### 2.2. Preprocessing

Prior to classification, neuroimaging and connectomic data must be appropriately preprocessed in order to allow efficient and precise analysis. Preprocessing can be divided into two steps, as outlined in the following sections: (1) preparation and quality assessment of neuro-images and extraction of time series data and (2) feature preprocessing.

#### 2.2.1. Preprocessing of Neuro-Images

As previously mentioned (see Section 2.1), the dataset used in this study consists of images collected from 17 different sites [21]. The standard preprocessing steps were taken to prepare the neuroimages for analysis using the Configurable Pipeline for the Analysis of Connectomes (CPAC) toolbox [10], which compiles preprocessing tools from multiple state-of-the-art neuroimaging analysis libraries, such as AFNI, FSL, ANTs, etc. Namely, the preprocessing frameworks in this pipeline include slice timing correction, motion correction, global mean intensity normalization and standardization of functional data to MNI space (3 × 3 × 3 mm resolution). We used data extracted with global signal regression and band-pass filtering (0.01–10Hz) in our analysis. After preprocessing was complete with the CPAC pipeline, ROI time series data was then extracted from the resulting fMRI images using the Automated Anatomical Labeling (AAL) functional parcellation brain atlas, which consists of N=116 labeled regions [19].

#### 2.2.2. Feature Preprocessing

As previously described in Section 3.1, connectomic data consists of the fMRI image scan, as well as the ROI time series data. While most state-of-the-art neuroimaging analysis studies focus on only one form of functional data, we computed both ROI connectivity matrices, i.e., ROI × ROI activation maps, and full-brain connectivity fingerprints, i.e., fMRI voxels × ROI activation maps, using the fMRI scan and ROI signal intensities.

**Activation map of fMRI and ROI.** The connectivity fingerprints between the functional images and mean time series data were computed by calculating correlation coefficients between every pair of voxels from the fMRI scan and ROI from the time series data [9]. We first restructured the 4D fMRI scan into a 2D matrix, such that each row of the matrix corresponds to one voxel, and each column is one intensity value at a given time T. The reshaped matrix would have thus have dimensions (H×W×D,T). We then compute a correlation matrix between the voxel intensities and the ROI time series data; the resulting matrix will be of dimensions (H×W×D,N), where *N* is the number of ROI.

**Figure 1:**
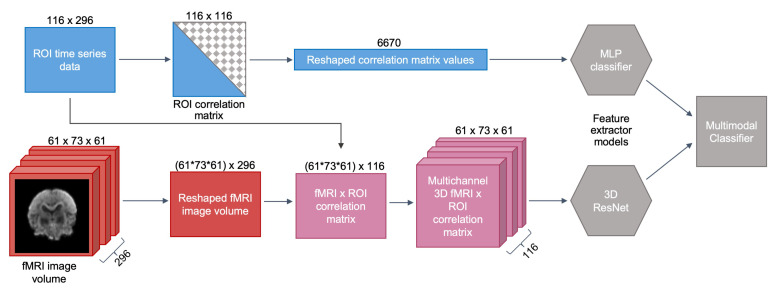
**Preprocessing workflow.** Construction of correlation matrices, or activation maps (terms are used interchangeably) between fMRI voxels × ROI time series and between pairs of ROI time series alone. Numbers on edges of the objects indicate matrix dimensions at the current step, e.g., the dimensions of the fMRI scan and ROI time series data are (H=61,W=73,D=61,T=296) and (N=116,T=296), respectively. For more detail on ResNet and MLP network architectures, refer to Figure 2.

**Activation map of only ROI.** The ROI functional connectivity maps were constructed by computing a correlation matrix for each subject, where each cell of the matrix contains the correlation coefficient between a pair of regions, i.e., the level of co-activation in the signals between any two given regions. Since the resulting correlation matrix is symmetrical along its diagonal, we removed the values in the lower triangle to delete the duplicates, and also deleted the main diagonal of the matrix, which represents the correlations of each region with itself. The remaining matrix values were then reshaped into a vector of features to use as input into the model. The number of features in the final vector is given by $\frac{1}{2}\left(\left(N-1\right)N\right)$, where *N* is the total number of regions [8]. Since the AAL brain atlas was used to extract the ROI time series data (N=116), the resulting vector consisted of 6670 features. The entire feature extraction and connectivity matrix construction workflow is illustrated in Figure 1.

### 2.3. Network Architectures

In this neuroimaging classification problem, the input to the model is an fMRI × ROI connectivity fingerprint and a vector of ROI correlation coefficients, and the goal is to diagnose the subject as either positive (with autism) or negative (healthy) based on both inputs. The proposed multimodal model extracts representations for both the fMRI scan and the ROI input, pools the feature vectors using simple vector concatenation, and arrives at a diagnosis using the combined features, which are propagated through four fully-connected layers before being classified into one of two classes.

We extracted features from fMRI × ROI correlation matrices using a modified 18-layer Residual Network (ResNet-18) [22]. All 2-dimensional convolution layers were replaced by their 3-dimensional equivalents in order to process the multichannel 3D correlation matrices. The correlation matrices were all uniformly of the dimensions (61,73,61,116), corresponding to (H,W,D,T) respectively. During training, the data is reshaped to be (116,61,73,61) so that the ROI dimension is used as the number of input channels into the first convolutional layer of the ResNet-18 network. Max-pooling is performed following the input convolutional layer over a 1×3×3 pixel window, with a stride of (1,2,2). The stride attribute determines how far the window or filter is moved in each dimension after every computation; e.g., with a stride of 2, the filter moves 2 pixels in the specified dimension. The matrix is then passed through a stack of convolutional blocks (Res-blocks), each with multiple convolutional layers, where we pass filters across the image to convolve over the height, width, and depth dimensions. The filters have very small receptive fields: 3×3×3 (which is the smallest size to capture the notion of left/right, up/down, center). In the first two convolutional blocks, we also use 1×3×3 or convolution filters. The convolution stride varies between 1 and 2 pixels depending on the dimension. Following the convolutional blocks, average pooling is performed over a 32×3×2 window, and the resulting output is a 512-D vector. The vector is then passed through a fully connected layer before returning the final model output.

ROI × ROI connectivity map features were extracted using a multilayer perceptron (MLP) classifier, which was a simple fully-connected network. The MLP consisted of three hidden layers, each with 100 nodes. As stated above, the ROI correlation matrices were reshaped into 6670-D vectors, where each element represents a pairwise correlation coefficient between the averageBOLD signal of two ROIs.

## 3. Experimental Details

**Figure 2:**
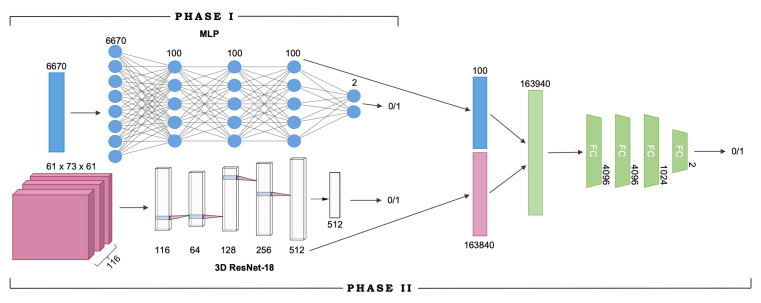
**Multimodal network architectures and training phases.** Visualization of the structure of the complete deep multimodal network. Numbers denote nodes or input channels in the labeled layer; FC implies fully connected layers. Phase I of training refers to the training of Resnet-18 and MLP for feature extraction; Phase II refers to the end-to-end training of the entire multimodal model after removing output layers from the feature extractors (as shown in the figure, the last 2-node layer of the MLP and the 512-channel layer of the ResNet are bypassed during Phase II of training.)

Training occurred in two separate phases due to the nature of the model architecture, as described in Section 4.2. In Phase I, the feature extractors were separately trained as independent networks. Namely, the MLP was trained on ROI activation maps, and ResNet-18 was trained on fMRI × ROI activation maps. Upon completion of training for both networks, Phase II of training began. Both MLP and ResNet-18 models were truncated to remove their output layers to be used as feature extractors, and then combined with the four fully-connected layers to form the end-to-end model. The resulting multimodal network, built from the pretrained MLP and ResNet-18 models (the learned network weights of both models were saved and loaded into the multimodal network), was trained from scratch in Phase II, initialized with the learned weights from Phase I. After training is complete, we report performance metrics of classification accuracy, precision, recall, and F1 score for each feature extractor model, as well as the final end-to-end multimodal model. The full network architecture and the two training phases are visualized in Figure 2.

### 3.1. Training Details

Prior to training, the neuroimaging datasets was randomly split into the training and testing sets. 900 subjects were used to train the model, and the remaining 135 subjects were used to evaluate the trained model. The same training and testing sets were used in both phases of training, i.e., to train both the feature extractor models and the overall combined multimodal network to avoid polluting the test set with training data, or vice versa.

The ResNet-18 network used in this work is detailed in Table 1. We used a rectified linear unit (ReLU) activation function [23] after each layer and Softmax cross-entropy loss to train the network using minibatch stochastic gradient descent. We used a batch size = 8 and momentum = 0.9 to train both the feature extractor models and the end-to-end multimodal model. Both the feature extractor models were trained from scratch. To train the 3D CNN feature extractor, we used a learning rate of $1\times 10^{-2}$ for 30 epochs and dropped the learning rate after every 6 epochs by a factor of $1\times 10^{-1}$; with the MLP, we used a learning rate of $1\times 10^{-2}$ across 50 epochs and dropped the learning rate by a factor of $1\times 10^{-1}$ after every 5 epochs. In the second phase of training for the end-to-end model, the feature extractor layers were initialized using the MLP and ResNet’s weights learned during Phase I of training; the entire network was then trained end to end with a learning rate of $1\times 10^{-5}$ for 30 epochs. The learning rate was dropped after every 8 epochs for this network with a factor of $1\times 10^{-1}$. All models were constructed using Pytorch [24] and were trained on a single Nvidia P6000 GPU. The end-to-end training process of both feature extractors and the complete multimodal model took roughly two hours.

We present details of the ResNet-18 network architecture in Table 1 above.

## 4. Results

Due to the absence of any standardized benchmarks or training-testing set splits, it is difficult to compare against other published works in this field. Previous papers also do not evaluate networks using the same metrics, and thus do not all report precision, recall and F1 scores, and thus cannot be directly compared with our results. The following studies use different architectures and do not specify the data used for training and testing, and are meant to serve only as a reference to the present paper.

Previous studies on functional imaging analysis used only one type of correlation matrix or activation map as input. Khosla et. al. [9] analyzed the fMRI voxel and ROI time series activation map, and achieved 73% accuracy with their ensemble model. Guo et. al. [16] conducted studies on the ROI time series activation map, and achieved 81% accuracy using a fully-connected DNN network. Heinsfeld et. al. [8], also only used the ROI time series activation map, and report classification accuracies ranging from 52% to 70% using DNNs. They also studied the same data using support vector machines and random forests, which achieved 62% and 58% accuracy, respectively.

When trained on a random subset of 90% of the total ABIDE-I dataset and evaluated on the remaining patients, our proposed multimodal model is able to achieve an accuracy of 74% and an F1-score of 0.805. More importantly, we are able to reach a very high recall of 95%, which is crucial for a computer-assisted diagnosis system—when physicians diagnose patients for disease, a false negative can have extremely adverse consequences.

### 4.1. Ablation Results

We compare the performance of the feature extractor models and the combined multimodal model in Table 2. The ResNet-18 network, trained on fMRI × ROI activation maps, achieved a classification accuracy of 73.1%; the MLP classifier, trained on ROI × ROI activation maps, achieved a classification accuracy of 70.8%. When the feature vectors from both networks are pooled and used in the combined multimodal model, the resulting network has a higher classification accuracy of 74%, as well as a very high recall of 95% and an F1 score of 0.805, indicating that integrating both types of input in the model improves the overall network performance and be able to more accurately predict the correct diagnosis.

### 4.2. Explanation of Model Predictions

**Figure 3:**
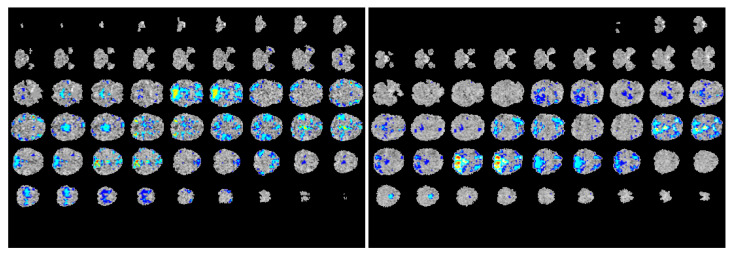
**Explanations of model predictions.** Cross-sections of fMRI scans indicating the regions that the network focused on when making predictions. Left: scans from a patient with autism; right: scans from a healthy individual with normal development. These images depict the regions the model used to predict the diagnosis of the scan, offering transparency on the model’s decision-making process. The visualizations were produced using techniques proposed by Selvaraju et al. [25].

Many medical imaging analysis techniques, especially those involving complex deep learning models, are difficult to interpret—it is difficult to decipher the inner workings of the networks or understand how they arrive at their predictions. To make our multimodal model more transparent, we visualized the regions of the fMRI images where the network focused on during the prediction process, as shown in Figure 3. Using gradient-weighted class activation mapping (Grad-CAM) as proposed by Selvaraju et. al. [25], we computed the activation gradients on the fMRI and ROI correlation matrices that were computed during the feature preprocessing stage of our study. We then overlaid these gradient-maps (heatmaps) on the original ABIDE fMRI scans to provide a clearer visual on which areas of the scan the model focuses on during the training and learning process. Through this visualization process, we were able to construct ‘visual explanations’ for the decisions made by our model by highlighting the important regions in the functional image for predicting the diagnosis. In doing so, we can clearly see the areas of the brain the model focuses on to identify differences between healthy control subjects and patients with autism. Moreover, this visualization will also aid doctors and physicians in the diagnostic process for ASD by assisting them in pinpointing the regions most likely to contain neural anomalies.

## 5. Conclusions

In this paper, we proposed a deep multimodal learning model for neuroimaging classification on the ABIDE dataset. Two types of functional imaging data, namely the activation maps calculated between fMRI image voxels × ROI time series and between only ROI time series, were used as input to two separate classifiers, a 3D ResNet-18 network and a multilayer perceptron classifier. The resulting feature vectors from these networks were pooled and used as input into several fully-connected layers to make the final prediction either control (healthy) or diseased (with autism). By proposing a model architecture that incorporates both types of activation maps, we use all the information provided by the functional data and demonstrate that the new model is capable of more accurate performance and thus makes for a better diagnosis system to assist doctors and medical practitioners.

However, at this point in time, the prediction accuracy of the multimodal model has only reached 74%, due to the limited amount of training data. This leads to the overfitting of our model.In the future, we plan to expand the training dataset by data augmentation and collecting more images to train our models in an effort to achieve higher accuracy. We hope that with further optimization and testing, we can eliminate any overfitting and reduce the amount of time needed for training. Moreover, we will be able to expand our analysis to other neuroimaging datasets, and demonstrate the generalizability of our methods by applying our automated system to diagnose more neurological diseases in addition to autism. With more testing and a larger quantity of data, we also hope to perform separate analysis on the visualizations of the model predictions, and to study the trends in which regions our model focuses on when making a diagnosis.

## Figures and Tables

**Table 1:** **ResNet-18 architecture.** Network architecture for 3D ResNet-18 model used for corr (fMRI, ROI) feature extraction. Building blocks are shown in brackets, with the numbers of blocks stacked.

Layer Name	3D ResNet-18	Output Size
conv1	5 × 5 × 5, 64, stride(2,2,2)	32 × 38 × 32 × 64
Max Pool	1 × 3 × 3 max pool, stride(1,2,2)	32 × 19 × 16 × 64
Res-block 1	$\left[\begin{array}{c} 1\times 3\times 3,64,\mathrm{stride}(1,1,1)\\ 1\times 3\times 3,64,\mathrm{stride}(1,1,1) \end{array}\right]$ × 2	32 × 19 × 16 × 64
Res-block 2	$\left[\begin{array}{c} 1\times 3\times 3,128,\mathrm{stride}(1,2,2)\\ 1\times 3\times 3,128,\mathrm{stride}(1,1,1) \end{array}\right]$ × 2	32 × 10 × 8 × 128
Res-block 3	$\left[\begin{array}{c} 3\times 3\times 3,256,\mathrm{stride}(1,2,2)\\ 3\times 3\times 3,256,\mathrm{stride}(1,1,1) \end{array}\right]$ × 2	32 × 5 × 4 × 256
Res-block 4	$\left[\begin{array}{c} 3\times 3\times 3,512,\mathrm{stride}(1,2,2)\\ 3\times 3\times 3,512,\mathrm{stride}(1,1,1) \end{array}\right]$ × 2	32 × 3 × 2 × 512
Average Pool	32 × 3 × 2 average pool	1 × 1 × 1 × 512
Fully connected	512 × 2 fully connected layer	2

**Table 2:** **Results and performance metrics.** Classification accuracy, precision, recall, and F1 scores for both feature extractor networks and final end-to-end multimodal model.

Training Phase	Input	Model	Accuracy	Precision	Recall	F1 Score
Phase I	corr(fMRI, ROI)	ResNet-18	0.731	0.726	0.849	0.783
	corr(ROI, ROI)	MLP	0.708	**0.737**	0.759	0.749
Phase II	**Both** corr	Multimodal	**0.740**	0.699	**0.949**	**0.805**

## Abbreviations

The following abbreviations are used in this manuscript:

MDPI	Multidisciplinary Digital Publishing Institute
DOAJ	Directory of open access journals
ABIDE	Autism Brain Imaging Data Exchange
CNN	convolutional neural network
Grad-CAM	gradient-weighted class activation mapping
ROI	regions of interest
